# Clinical impact of stellate ganglion phototherapy on ventricular tachycardia storm requiring mechanical circulatory support devices: a case report

**DOI:** 10.1093/ehjcr/ytae177

**Published:** 2024-04-30

**Authors:** Kei Takahashi, Yasuyuki Egami, Masami Nishino, Jun Tanouchi

**Affiliations:** Division of Cardiology, Osaka Rosai Hospital, 3-1179, Nagasone-cho, kita-ku, Sakai, Osaka 591-8025, Japan; Division of Cardiology, Osaka Rosai Hospital, 3-1179, Nagasone-cho, kita-ku, Sakai, Osaka 591-8025, Japan; Division of Cardiology, Osaka Rosai Hospital, 3-1179, Nagasone-cho, kita-ku, Sakai, Osaka 591-8025, Japan; Division of Cardiology, Osaka Rosai Hospital, 3-1179, Nagasone-cho, kita-ku, Sakai, Osaka 591-8025, Japan

**Keywords:** Ventricular tachycardia storm, Stellate ganglion phototherapy, Mechanical support devices, Near-infrared radiation therapy, Old myocardial infarction, Case report

## Abstract

**Background:**

Ventricular arrhythmias are a significant cause of morbidity and mortality in patients with ischaemic heart disease. When pharmacologic therapies, catheter ablation (CA), and implantable cardioverter defibrillator (ICD) are ineffective, stellate ganglion blockade (SGB), sympathectomy, and renal sympathetic denervation are considered. However, they are invasive for patients with high bleeding risk. We present a case of successfully recovering from haemodynamically unstable ventricular tachycardia (VT) storm with stellate ganglion phototherapy (SGP) in a non-invasive manner.

**Case summary:**

A 73-year-old male presented to the emergency department with chief complaint of general malaise, resulting from VT storm associated with ischaemic cardiomyopathy. He had a history of CA and implantation of ICD. Despite multiple electrical cardioversions, pharmacologic therapies, and deep sedation with mechanical circulatory support (MCS), VT storm was not controlled. Thereafter, we irradiated the patient’s neck with SGP to inhibit sympathetic neurological activity, which suppressed VT storm and dramatically improved his haemodynamic status.

**Discussion:**

It has been reported that SGP is an alternative to SGB for refractory VT storm. Stellate ganglion phototherapy was easy and non-invasive to perform because we just irradiated the patient’s neck with the near-infrared light for 5 min per day. If conventional therapies are ineffective in suppressing VT storm, SGP may be considered as a next step, especially for patients with high bleeding risk. However, since the effect of a single SGP lasts only 1–2 days, it should be performed as a bridge therapy to CA or sympathectomy. Stellate ganglion phototherapy may be effective in suppressing VT storm that requires MCS devices.

**Learning points**

- Ventricular tachycardia (VT) storm has a significant impact on patient quality of life and prognosis. Pharmacologic therapies, catheter ablation (CA), and implantable cardioverter defibrillator implantation are standard management to suppress VT storm. However, these therapies are occasionally ineffective in haemodynamically unstable VT storm.
- Stellate ganglion blockade, sympathectomy, and renal sympathetic denervation are well-known as alternative treatments, but they have a risk of bleeding complication.
- Stellate ganglion phototherapy (SGP) may be considered as a next step, especially for patients with high bleeding risk. This may be particularly useful in patients with polymorphic ventricular tachycardia since CA is less likely to be successful if the VT is not monomorphic and also may have a role in ventricular arrhythmias that are not scar-dependent.

We present a case of successfully recovering from haemodynamically unstable VT storm with SGP in a non-invasive manner.

## Introduction

Ventricular arrhythmias (VAs) are a major cause of morbidity and mortality in patients with ischaemic heart disease.^1^ The autonomic nervous system is known to play a role in the genesis and maintenance of VAs.^2^ Modulation of the autonomic nervous system is effective for ventricular tachycardia (VT) storms. When conventional therapies such as medications and catheter ablation (CA) are ineffective in suppressing VT storms, a stellate ganglion blockade (SGB), sympathectomy, and renal sympathetic denervation emerge as alternative therapies. However, those therapies are invasive and may increase bleeding complications in patients at risk for bleeding. Recently, Nonoguchi *et al.*^3^ reported that stellate ganglion phototherapy (SGP) with a low-level laser is effective in controlling VAs without bleeding complications. However, the efficacy of SGP for refractory VT storms requiring mechanical circulatory support (MCS) devices remains unknown. We present a case in which we successfully controlled VT storm with SGP in a non-invasive manner, which had been refractory to conventional treatments and required MCS devices.

## Summary figure

After administration of enough medical therapy and MCS devices insertion, the frequency and duration of VT storm gradually decreased. On the 4th hospital day, VT storm disappeared transiently but reoccurred and persisted again on Day 5. Then, we decided to perform stellate ganglion phototherapy (SGP) with Super Lizer from Day 6 considering the high bleeding risk. We irradiated his left-sided neck with near-infrared light for 5 min per day directly without ultrasound. The target of irradiation was set between the cricoid cartilage and the anterior border of the sternocleidomastoid muscle that located the jugular groove ∼2.5 cm above the costoclavicular joint. From the first attempt of the SGP, the duration of VT storm decreased gradually and disappeared completely. As his haemodynamics improved since Day 7, he was successfully weaned from ECMO on the same day. Despite removing MCS, VT storm was suppressed and his haemodynamics was kept in a stable condition. Finally, he left the ICU on Day 17. Since the daily SGP effectively controlled the VT episodes, a sympathectomy was scheduled on Day 54. However, the patient unexpectedly died on Day 52 due to a VT storm.

**Figure:**
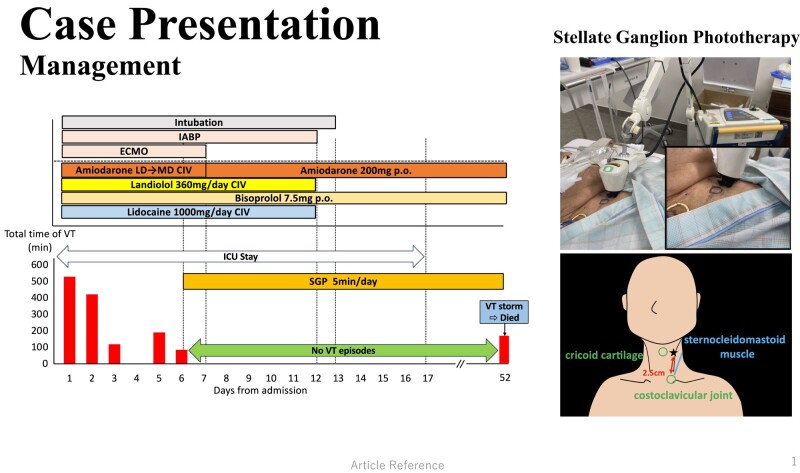


## Case presentation

**Figure 1:**
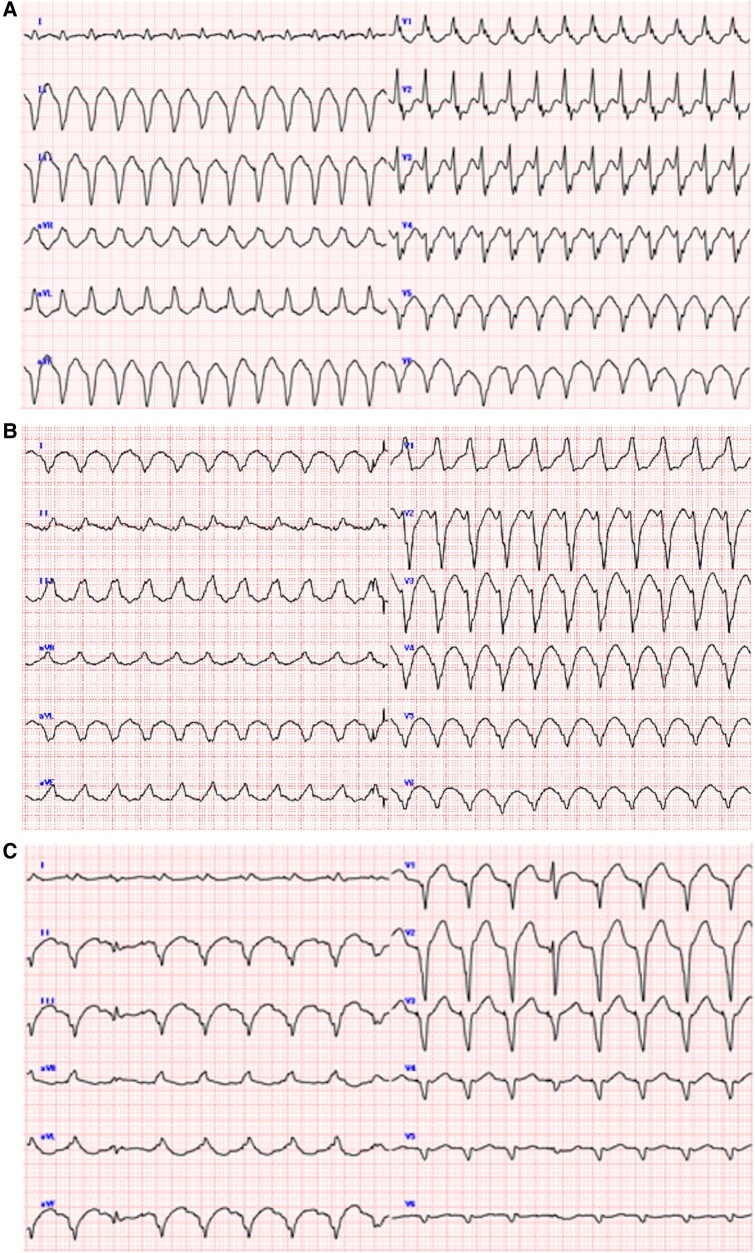
(*A*) Left bundle branch block morphology and inferior axis with a QS in lead I. (*B*) Right bundle branch block morphology and superior axis with a QS in lead I. (*C*) Right bundle branch block morphology and superior axis with a qR in lead I. ECG: polymorphic VT storm. VT, ventricular tachycardia.

**Figure 2:**
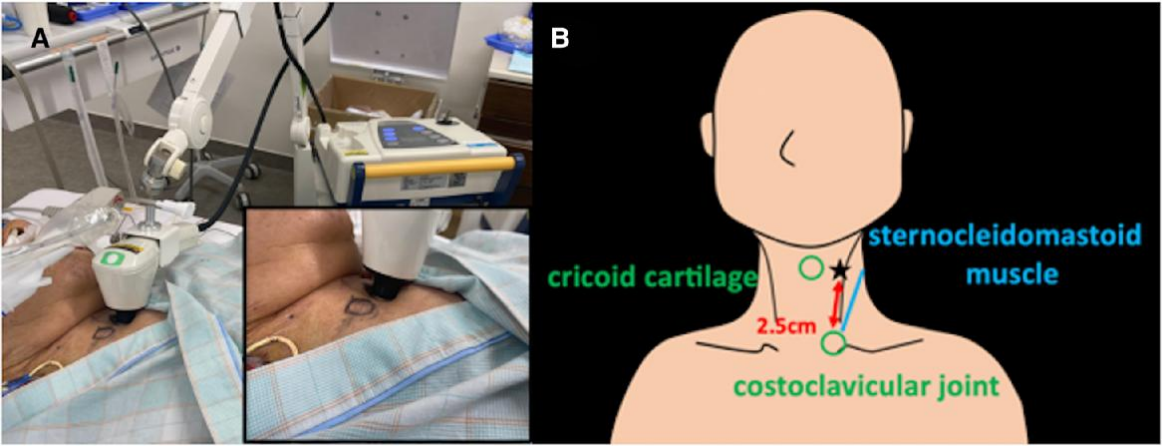
NIRT for the LSG using a Super Lizer (Tokyo Iken, Japan Co., Ltd). Irradiation towards the patient’s neck on the left side between the cricoid cartilage and anterior border of the sternocleidomastoid muscle (the jugular groove ∼2.5 cm above the costoclavicular joint). NIRT, near-infrared radiation therapy; LSG, left stellate ganglion. (*A*) The patient received NIRT, (*B*) the target (black star) of the stellate ganglion phototherapy.

**Figure 3:**
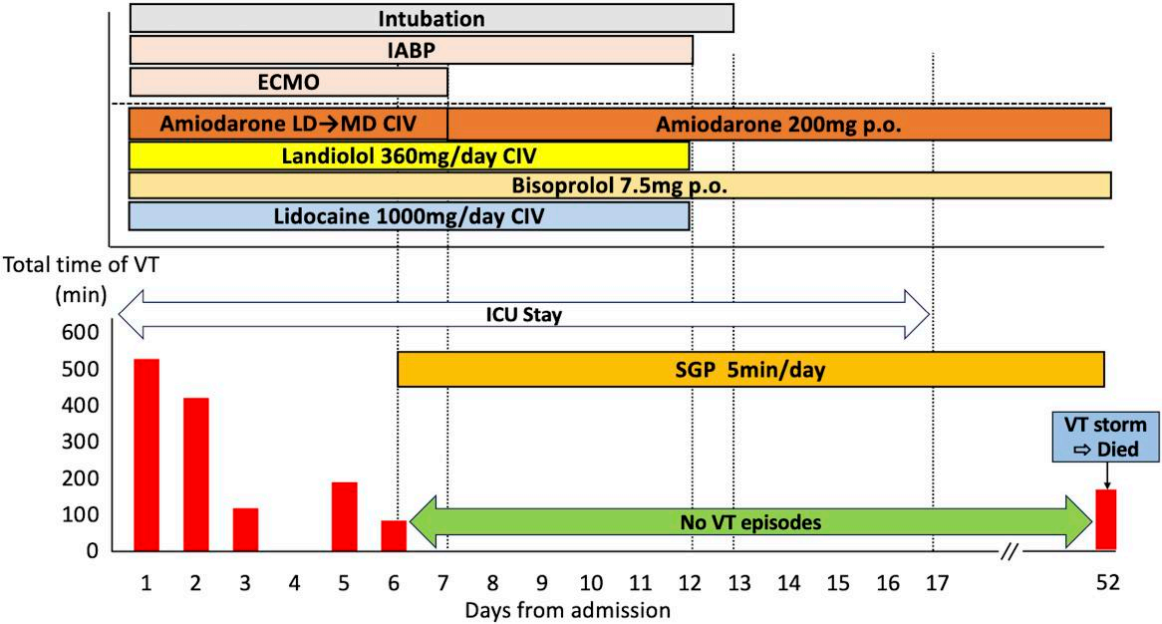
Clinical time course of before and after SGP. Medical therapies, including amiodarone, landiolol, bisoprolol, and lidocaine, and deep sedation with mechanical support devices (ECMO and IABP) were insufficient to control VT storm. After initiation of SGP on the left side neck for 5 min daily, VT storm was completely suppressed until Day 51. Following the stabilization of the patient’s haemodynamics, the mechanical support devices were successfully removed without any complication and he was discharged from the ICU on Day 17. Nonetheless, on Day 52, the VT storm recurred and he died.’ CIV, continuous intravenous infusion; ECMO, extracorporeal membrane oxygenation; IABP, intra-aortic balloon pumping; ICU, intensive care unit; LD, loading dose (125 mg for 10 min and 288 mg for 6 h); MD, maintenance dose (600 mg/24 h); P.O., per OS; SGP, stellate ganglion phototherapy. The other abbreviations are shown in *Figure 1*.

A 73-year-old male with ischaemic cardiomyopathy and a reduced left ventricular ejection fraction of 30% was brought to our hospital with a complaint of palpitation and general malaise. Previously, he underwent aortic valve replacement with a mechanical valve and coronary artery bypass grafting. Later, he experienced sustained ventricular tachycardia and underwent unsuccessful catheter ablation. Subsequently, he underwent implantation of an implantable cardioverter defibrillator (ICD). Before the current admission, he was stabilized medically with bisoprolol 7.5 mg, azosemide 30 mg, spironolactone 25 mg, rosuvastatin 5 mg, lansoprazole 15 mg, warfarin 1 mg, empagliflozin 10 mg, febuxostat 10 mg, and amiodarone 100 mg. On arrival, the 12-lead electrocardiogram during the VT showed three distinct morphologies (*Figure 1A*–*C*). Despite multiple attempts of electrical cardioversion, the VT storm persisted leading to a decline in his haemodynamic stability. He was hospitalized for recurrent haemodynamically unstable VTs. Subsequently, he required intubation with sedatives and MCS devices [extracorporeal membrane oxygenation (ECMO) and intra-aortic balloon pumping (IABP)]. Intravenous administration of amiodarone (loading dose: 125 mg for 10 min and 288 mg for 6 h, maintenance dose: 600 mg/24 h), landiolol (360 mg/24 h), and lidocaine (1000 mg/24 h) was initiated as the primary therapeutic regimen. The frequency and duration of the VT storms gradually decreased, albeit the transient disappearance on Day 4, followed by its reappearance and persistence on Day 5. Because at least three different morphologies of the VT were identified despite the previous VT ablation, we concluded that the elimination of those VTs by a re-ablation would be very difficult. Then, a SGB was considered as an alternative therapy. However, it was deemed inadequate due to the high risk of bleeding, given that the patient required anticoagulant therapy involving the continuous administration of heparin for the MCS devices and the aortic prosthetic valve. Therefore, SGP with the Super Lizer (Tokyo Iken, Japan Co., Ltd), which uses near-infrared light with a wavelength of 600–1600 nm, was selected. From Day 6, a daily exposure of 5 min of near-infrared light targeting his left-sided neck, specifically between the cricoid cartilage and the anterior border of the sternocleidomastoid muscle (the jugular groove ∼2.5 cm above the costoclavicular joint, *Figure 2A* and *B*) was performed. From the first attempt of the SGP, the duration of the VT storm decreased gradually and disappeared completely. Consequently, with an improvement in his haemodynamics from Day 7, he was successfully weaned from ECMO on the same day. Despite the ECMO removal, the VT storms remained controlled, maintaining his haemodynamic stability. This allowed for the removal of the IABP on Day 12, followed by extubating on Day 13. Finally, he was discharged from the ICU on Day 17 (*Figure 3*). Upon transfer to the general ward, the patient was offered the option of a re-do catheter ablation or hybrid surgical ablation, but he declined any further cardiac interventional therapy. He agreed to the SGP and provided written informed consent. Since the daily SGP effectively controlled the VT episodes, a sympathectomy was scheduled on Day 54. However, the patient unexpectedly died on Day 52 due to a VT storm.

## Discussion

Ventricular tachycardia storms delineate a condition characterized by the clustering of ventricular arrhythmic events, significantly impacting the patient’s quality of life and long-term prognosis.^4^ The conventional management of VT storms includes the administration of medications like amiodarone and beta-blockers, deep sedation, CA, and ICD implantations.^5^ However, these treatments may occasionally prove insufficient to control VT storms, as observed in this case. Modulation of the autonomic nervous system has been shown to be effective in suppressing VT storms. An SGB, sympathectomy, and renal sympathetic denervation are also well-known as alternative treatments. The stellate ganglion, which is the therapeutic target of neuromodulation therapies, is located in the intervertebral space between the C7 and T1 vertebral bodies. Meng *et al.*^6^ reported that the SGB is an effective acute treatment for electrical storms. The SGB reduced the burden of arrhythmia and defibrillation events 24 and 48 h after a nerve blockade.^7^ However, in our case, the SGB was contraindicated due to the administration of anticoagulant therapy, specifically heparin. Notably, according to the guidelines,^8^ an SGB during anticoagulant therapy is classified into a high-risk procedure, supported by several reports highlighting the potential for bleeding complications.^9^ Instead, SGP has become a widely used alternative therapy for the treatment of chronic pain and hyperhidrosis.^10^ Yoshida *et al.*^11^ reported that SGP with a near-infrared radiation therapy to block the sympathetic activity has a similar effect as the SGB in managing VAs. This non-invasive method involves irradiation through the skin surface using a phototherapy treatment device: Super Lizer (Tokyo Iken Co), which uses linearly polarized near-infrared light within a 600–1600 nm wavelength band, effectively inhibiting the sympathetic neurological activity without any other nerve blockade.^3^ Additionally, Fulop *et al.*^12^ revealed its anti-inflammatory effects, sympathetic tone inhibition, and vasodilatation effects. Saeki *et al.*^13^ demonstrated that SGP does not accompany pain and rarely shows any side effects such as Horner’s syndrome and a vagal or laryngeal recurrent nerve blockade. In addition, SGP remains suitable even for patients undergoing anticoagulant therapy or experiencing bleeding disorders. Given these studies suggesting SGP’s non-invasive and effective potential in suppressing VT storms, we decided to apply SGP in this patient. Although a VT ablation was considered, concern about the potential adverse effects on his cardiac function and the risk of thrombus formation led to the decision not to perform a VT catheter re-ablation. Moreover, we anticipated difficulties in mapping and ablating the multiple and polymorphic VTs. Thus, we decided against performing a VT re-do catheter ablation. Stereotactic body radiotherapy (SBRT) has emerged as a safe and effective therapy for refractory VTs.^14^ However, in our case, SBRT was not preferable given the risks associated with moving the patient with two mechanical devices and a ventilator to the SBRT room. Conversely, SGP had the advantage of the patient’s bedside therapy, rendering it more suitable for the patient than SBRT. Additionally, Nonoguchi *et al.*^3^ demonstrated the efficacy of SGP using a low-level laser in reducing the sympathetic activity for refractory VAs. In their study, the enrolled patients had frequent episodes of VAs despite pharmacologic therapy and/or CA, but they were haemodynamically stable and did not receive any MCS devices, unlike our case. Moreover, they received irradiation for 10 min daily towards the bilateral neck in the supine position with probes firmly placed between the cricoid cartilage and the anterior border of the sternocleidomastoid muscle. In contrast, our approach involved irradiating solely the left side of the patient’s neck for 5 min daily with the SGP. Sato *et al.*^15^ demonstrated that continuous irradiation of only one side of the patient’s neck for 5 min with SGP may serve as an adjunctive therapy or bridge therapy for refractory VAs. Therefore, we also adopted a 5 min SGP therapy. Notably, SGP exclusively on the left side for 5 min daily demonstrated a significant efficacy in suppressing VT storms. Therefore, if conventional therapies fail to suppress VT storms, especially in patients with a high bleeding risk, SGP may be considered as an alternative therapy. However, due to the transient effect lasting only 1–2 days after the SGP, it may serve as a bridge therapy until a CA or stellate ganglion sympathectomy is performed. Finally, in our case, the patient unexpectedly died on Day 52 due to a VT storm. Weaning off the antiarrhythmics might be the possible precipitating factors of the recurrence of VT storm, but we had to switch intravenous administration of these drugs to oral medicine.

## Data Availability

The data underlying this article will be shared on reasonable request to the corresponding author.

## Lead author biography

Dr Kei Takahashi graduated from Osaka University in 2018. He completed his residency programme at Osaka Rosai Hospital and Kansai Rosai Hospital. His areas of interest are percutaneous coronary intervention, endovascular therapy, and transcatheter aortic valve replacements.

**Consent:** The authors confirm that written consent for the submission and publication of this case report, including the accompanying images and associated text, has been obtained from the patient in line with COPE guidelines.

**Funding:** None declared.
